# Probing lipid membrane bending mechanics using gold nanorod tracking

**DOI:** 10.1103/physrevresearch.4.l012027

**Published:** 2022-03-07

**Authors:** Mehdi Molaei, Sreeja Kutti Kandy, Zachary T. Graber, Tobias Baumgart, Ravi Radhakrishnan, John C. Crocker

**Affiliations:** 1 Chemical and Biomolecular Engineering, University of Pennsylvania, Philadelphia, Pennsylvania 19104, USA; 2 Bioengineering, University of Pennsylvania, Philadelphia, Pennsylvania 19104, USA; 3 Chemistry, University of Pennsylvania, Philadelphia, Pennsylvania 19104, USA

## Abstract

Lipid bilayer membranes undergo rapid bending undulations with wavelengths from tens of nanometers to tens of microns due to thermal fluctuations. Here, we probe such undulations and the membranes’ mechanics by measuring the time-varying orientation of single gold nanorods (GNRs) adhered to the membrane, using high-speed dark field microscopy. In a lipid vesicle, such measurements allow the determination of the membrane’s viscosity, bending rigidity, and tension as well as the friction coefficient for sliding of the monolayers over one another. The in-plane rotation of the GNR is hindered by undulations in a tension dependent manner, consistent with simulations. The motion of single GNRs adhered to the plasma membrane of living cultured cells similarly reveals the membrane’s complex physics and coupling to the cell’s actomyosin cortex.

The plasma membrane of cells serves as a boundary and mediates force transmission and information and material flow between inside and outside, via cytoskeletal adhesion proteins, signal receptor proteins, and endocytic and exocytic vesicle formation. This fluidic lipid membrane displays time-dependent local curvature or undulations, which are controlled by its bending modulus, excess area, and membrane tension. The complex physics of model lipid membranes, as in giant unilamellar vesicles (GUV) formed of purified lipids, is relatively well understood, having been studied by a variety of techniques [1–9]. In contrast, the physical properties and dynamics of the plasma membrane of cells are notoriously difficult to measure [10–15], due largely to the membrane being attached to the underlying viscoelastic actin cortex by adhesion proteins—deformations and undulations on long length scales are those of the composite membrane-cortex structure. The mechanics of the plasma membrane are relevant for understanding vesicle trafficking and membrane blebbing and tension sensing as well as the modulation of membrane protein binding and assembly.

Here we utilize orientational tracking of single gold nanorods (GNRs) to characterize the bending dynamics of reconstituted lipid GUVs and the plasma membrane of cultured cells. While the in-plane rotation of anisotropic probes has previously been used to infer membrane viscosity [16], we exploit three-dimensional orientational tracking to follow membrane bending modes. In GUVs, we use a model for the out of plane angular motion of GNRs to determine properties of the lipid bilayers including their bending modulus, tension, and intermonolayer friction coefficient. The complex motion of a nanorod on an undulating membrane is also studied through Monte Carlo simulation. The angular motion of GNRs bound to a Huh7 cell is tracked to investigate bending dynamics of a plasma membrane.

To prepare nanorod probes that bind GUVs and cell plasma membranes, we started with carboxyl-modified GNRs (Nanopartz) having nominal length *l* = 140 ± 20 nm and diameter 2*a* = 40 ± 4 nm and covalently attached magainin 2 peptides (Genscripts) to them using a water-soluble carbodiimide protocol [17]. After washing out excess peptide by centrifugation, small amounts of suspended GNRs were mixed with either a GUV suspension and loaded into microscopy chambers or added to cells cultured on cover-slip bottom petri dishes. GUVs were formed by drop casting mixed lipids (Avanti) dissolved in an organic solvent on indium tin oxide (ITO) coated slides, vacuum drying to completely remove the solvent followed by electroformation [18,19] in an aqueous buffer containing 0.3 M sucrose as an osmolyte. Huh7 cells (ATCC) were cultured in petri dishes using standard growth medium containing antibiotics, and grown to ≈50% confluence. This medium was exchanged for pH = 7.3 PBS containing GNRs for imaging, performed at room temperature. For additional details see the Supplemental Material (SM) [20].

We track the translational and rotational motion of single GNRs using a custom-built polarimetric dark field microscope, using methods reported in an earlier study [23]. Briefly, this instrument epi-illuminates the GNR with circularly polarized, collimated laser light (*λ* = 670 nm), collects the backscattered light from the GNR, and forms it into two images having orthogonal linear polarizations. The translational position *x* and *y* of the GNR over time *t* is determined by particle tracking [24,25]. This is then mapped relative to the GUV’s center coordinates, *x*_*c*_, *y*_*c*_, and radius, *R*, determined using phase contrast microscopy. The total brightness of the GNR in the two polarization channels allows the reconstruction of the rod’s orientation in 3D space to a precision of about 1 °, at several thousand measurements per second. The symmetry of this method, however, projects the actual orientation to a $\hat{u}(t)$ in one octant of the full sphere [23]. The measured mean-squared angular displacement (MSAD), $\langle \Delta {\hat{u}}^{2}(\tau )\rangle ={\langle {\left[\hat{u}(t+\tau )-\hat{u}(t)\right]}^{2}\rangle }_{t}$, where ⟨·⟩ denotes a time average, is thus bounded unphysically at long times. These “raw” MSADs can be reliably converted into their corresponding physical (unbounded) MSADs, ${\langle \Delta {\hat{u}}^{2}(\tau )\rangle }_{l}$, however, using an algebraic mapping. Only the physical MSADs are reported and analyzed below. Details of the instrument and GNR tracking are provided in the SM [20].

We first prepare GUVs with different lipid compositions and different membrane tensions, and compare the GNRs translation and rotational motion on them to model predictions. Specifically, to vary membrane viscosity *η*_*m*_ and bending modulus *κ*_*c*_, we form GUVs with two different compositions, having either 1:1 composition of 1,2 dioleoylsn-glycero-3-phosphocholine (DOPC) and 1,2-dioleoyl-sn-glycero-3-phospho-L-serine (DOPS) or 1:1:1 composition of DOPS-DOPC-cholesterol. We also change the effective membrane tension, *σ*, of the cholesterol GUVs by changing the buffer solution. Suspending the GUVs in isotonic buffer leaves them “floppy” (having excess membrane area), while slightly hypotonic buffer leads them to swell and become “tense,” (reducing their excess area).

The experimental 3D trajectories of single GNRs over time *t*, ***r***(*t*) = [*x*(*t*) − *x*_*c*_, *y*(*t*) − *y*_*c*_, *z*(*t*)] diffusing at different locations on the no-cholesterol GUV are shown in Fig. 1(a), where *z* = [*R*^2^ − (*x* − *x*_*c*_)^2^ − (*y* − *y*_*c*_)^2^]^1*/*2^ is the depth of the GNR. The random motions in all three dimensions are Gaussian distributed [20, Fig. S1]. The coordinates of the nanorod orientation and membrane normal are shown in Fig. 1(b) and distribution of rod orientations are shown in Fig. 1(c), mapped into an octant of the unit sphere. The translational mean-squared displacement (MSD) of GNRs, ⟨Δ***r***^2^(*τ*)⟩ = ⟨[***r***(*t* + *τ* ) − ***r***(*t*)]^2^⟩_*t*_, on different GUVs are shown in Fig. 1(d). The MSD of a particle diffusing over the surface of a sphere is bounded at long times [20], but satisfies ⟨Δ***r***^2^(*τ*)⟩ = 4*D*_*t*_*τ* for *τ* ≪ *R*^2^*/D*_*t*_, where *D*_*t*_ = *k*_*B*_*T/γ*_*t*_ is the translational diffusivity and *γ*_*t*_ and *k*_*B*_*T* are the translational drag coefficient and thermal energy. Similarly, the mean-squared angular displacement (MSAD), ${\langle \Delta {\hat{u}}^{2}(\tau )\rangle }_{l}$, of the GNRs is used to determine the in-plane rotational drag coefficient of the rods, *γ*_*r*_. For rotational diffusion of a rod lying prone in a plane (an orientation that maximizes their adhesion energy), the MSAD satisfies ${\langle \Delta {\hat{u}}^{2}(\tau )\rangle }_{l}=4D_{r}\tau$, where *D*_*r*_ = *k*_*B*_*T/γ*_*r*_ is the rotational diffusivity and *γ*_*r*_ is the rotational drag coefficient.

Fitting the measured MSDs and MSADs yields diffusivities *D*_*t*_ and *D*_*r*_ for single GNRs and their drag coefficients *γ*_*t*_ and *γ*_*r*_ on different GUVs. In general, these drag coefficients depend on the membrane viscosity, *η*_*m*_, the effective size of the nanorod, and the bulk fluid viscosity *η* [16,26]. For the GNR on the tense GUV without cholesterol, we obtain *D*_*t*_ = 0.52 ± 0.06 μm^2^*/*s and *D*_*r*_ = 27.7 ± 3.1 rad^2^*/*s. Using a model for *γ*_*t*_ and *γ*_*r*_ [27] and a bulk fluid viscosity *η* = 1 mPa s [20], we find the membrane viscosity *η*_*m*_ = 1.2 ± 0.1 nPa s m and the effective length [16] of the GNR *l*_*eff*_ = 202 ± 25 nm. The value of membrane viscosity is in good agreement with literature values [16,28–30]. Such values imply that the Saffman-Delbrük length scale, set by the ratio of the membrane and bulk viscosities, *l*_*m*_ = *η*_*m*_*/η* ≈ 1 μm, is an order of magnitude larger than the GNR length, ensuring that its motion is dominated by membrane mechanics rather than the bulk fluid [16].

The membrane viscosity of the tense GUV with cholesterol is estimated as *η*_*m*_ = 17.7 ± 0.2 nPa s m, which is an order of magnitude larger than *η*_*m*_ of the no-cholesterol GUV. The effect of cholesterol on the fluidity of lipid membranes depends on temperature [31] and lipid composition [32]. While cholesterol increases the fluidity of lipid membranes with saturated or monounsaturated fatty acids tails [32], it raises the viscosity of unsaturated tail lipids, such as DOPC-DOPS here, by about one order of magnitude [33]. Interestingly, the MSAD of the GNR on the floppy GUV is lower than the same composition tense GUV and shows subdiffusive behavior [Fig. 1(e)], which we argue below is due to transient caging by membrane undulations.

The next, and major goal of this study is to quantify the membrane bending dynamics by measuring the time-dependent out of plane random motion of the GNRs. Specifically, while a GNR is diffusing on a GUV, the membrane of the GUV is also undergoing thermal undulation that causes tilting (or out of plane) motion that can be measured. We estimate the membrane normal direction $\hat{n}$ by fitting the time-dependent nanorod orientation over a few measurements to a plane, as illustrated in Fig. 1(b), using singular value decomposition [20, Fig. S5(a)]. This approach relies on the fact that in-plane rotational diffusion is typically much faster than the corresponding diffusion of the membrane normal, and is validated using Monte Carlo simulation [20, Fig. S5(b)].

The dynamic “out of plane” angle is computed between the normal vector and the expected normal if the GUV were a perfect sphere: $\theta =\cos^{-1}\hat{n}\cdot \hat{r}$. The MSDs of this out of plane motion ⟨Δ*θ*^2^(*τ*)⟩, shown in Fig. 2, reveal dramatic differences in the undulation dynamics among different GUVs. Membrane undulatory dynamics has often been studied by analyzing the bending fluctuations of membranes, for example, using optical microscopy. In the long-wavelength (small wave number, *q*) limit, in the Monge gauge, the spectrum of the height undulations of an elastic membrane varies as ⟨*h*_*q*_*h*_*−q*_⟩ ~ 1*/q*^4^. Since angular fluctuations correspond to the slope or derivative of the undulation height function, we expect them to vary as ⟨*θ*_*q*_*θ*_*−q*_⟩ ~ 1*/q*^2^ [20]. This suggests that angular measurements, as in our experiment, are far more sensitive to higher wave numbers than direct height measurements, improving our measurement sensitivity to small amplitude, submicron wavelength undulations.

Undulations with different wavelengths relax at different rates, so the lag-time dependence of the normal fluctuations provides spectral information about the amplitude of undulations at different wavelengths. Previous measurements of membrane fluctuations at high wave number, such as small-angle x-ray scattering and neutron spin echo (NSE) spectroscopy [34–37], have revealed departures from the classical picture of a membrane as a thin fluid sheet coupled to a viscous bulk fluid [38]. At length scales closer to the membrane thickness, *d*, membrane viscosity and the friction of sliding between the two monolayers act as additional sources of dissipation and lead to a larger effective dynamic bending modulus than that observed in the long wavelength regime [39]. Studies using MD simulation [40–42] and high-speed video microscopy [43] have elucidated the relaxation rates for these undulations in various *q* regimes. Adapting the Seifert-Langer model for the height correlation function [39], we develop a model for out of plane rotational motion of a GNR [20]:

(1) $$\langle \Delta \theta^{2}(\tau )\rangle =\frac{k_{B}T}{\pi }\int_{q_{\min}}^{q_{\max}}\frac{qdq}{\kappa_{c}q^{2}+\sigma }[1-\frac{\kappa_{c}}{{\tilde{\kappa }}_{c}}e^{-\omega_{2}\tau }$$

(2) $$-\left(1-\frac{\kappa_{c}}{{\tilde{\kappa }}_{c}}\right)e^{-\omega_{1}\tau }]+{\langle \Delta \theta^{2}\rangle }_{\text{static}},$$

where *q*_min_ = *π/R* and *q*_max_ = 2*π/l* are the minimum and the maximum wave numbers set by the size of the GUVs and the GNRs, respectively, *σ* is the tension, *κ*_*c*_ is the bending rigidity, and ⟨Δ*θ*^2^⟩_static_ is the measurement error. Here, ${\tilde{\kappa }}_{c}=\kappa_{c}+2\varepsilon d^{2}$ is the renormalized bending rigidity accounting for the effect of the elastic stretching and compression, where *ε* is the compressibility modulus of the membrane. The relaxation rates *ω*_1_*, ω*_2_ depend on *q*, the above parameters, and the friction coefficient between layers, *b*, as described in [20].

The asymptotic value of the out of plane displacement is controlled solely by bending rigidity and tension,

(3) $$\langle \Delta \theta_{\infty }^{2}\rangle =\frac{k_{B}T}{2\pi \kappa_{c}}\ln\frac{\kappa_{c}q_{\max}^{2}+\sigma }{\kappa_{c}q_{\min}^{2}+\sigma }.$$

We use the variance of the measured *θ* across multiple measurements to estimate $\langle \Delta \theta_{\infty }^{2}\rangle$, considering ⟨Δ*θ*^2^(*τ*)⟩ = 2⟨*θ*^2^(*t*)⟩_*t*_ − 2⟨*θ*(*t*)*θ*(*t* + *τ*)⟩_*t*_ and that the correlation function decays to zero at long time. Thus Eq. (3) provides an additional relationship between *κ*_*c*_ and *σ*.

To determine the lipid membrane properties using the out of plane motion of an attached nanorod, such as in Fig. 2, we simultaneously fit Eq. (1) and Eq. (3). To limit the number of free parameters in the model, we choose nominal values from the literature for the compressibility modulus *ε* = 0.1 Nm^−1^ and lipid layer thickness *d* = 2 nm [43].

For the GUV with no cholesterol, we find *b* = 6.9 ± 2.0 × 10^8^ Ns*/*m^3^, *κ*_*c*_ = 7.3 ± 1.1*k*_*B*_*T*, and *σ* = 1.1 ± 0.5 × 10^−6^ Nm^−1^. We confirmed the sensitivity of the measurement to the fitting parameters by performing a *χ*^2^ test [20, Fig. S7]. The uncertainties reported here take into account the uncertainty in the GNR length which sets the value of *q*_max_ [20, Fig. S8] and contributions from fitting uncertainty. To estimate uncertainty in *σ*, we also consider the standard error in ⟨*θ*^2^(*t*)⟩ determined from more than 10 GNR trajectories with time spans of more than 2 min between each measurement. The values obtained from the fitting agree well with the ranges reported in the literature for *b* = 0.5–5 × 10^8^ Ns*/*m^3^ [43–45] and *κ*_*c*_ ≈ 10*k*_*B*_*T* [43,46–49]. Adding cholesterol to the lipid composition roughly doubles *b* = 1.2 ± 0.5 × 10^9^ Ns*/*m^3^ and triples *κ*_*c*_ = 19.2 ± 0.8*k*_*B*_*T* consistent with literature results [43]. The membrane properties inferred from three replicates of the tense GUV case fall within the uncertainty range [20, Fig. S9 and Table S.1]. As expected, the floppy GUV shows a low surface tension *σ* = 5.1 ± 8.4 × 10^−8^ Nm^−1^ (comparable to our tension detection limit), while the tense GUV of the same composition shows a roughly 100× larger value: *σ* = 4.5 ± 1.3 × 10^−6^ Nm^−1^.

To better understand the complex kinematics of a nanorod moving on membranes with different tensions, we constructed a model GNR-membrane system [20, Fig. S10] based on the dynamically triangulated Monte Carlo (DTMC) method [50]. This model uses a continuum approximation of the membrane based on the Canham-Helfrich Hamiltonian $H_{\text{elastic}\;}=\int ds\left[\kappa_{c}{\left(c_{1}+c_{2}\right)}^{2}/2\right]$, where *c*_1_ and *c*_2_ are the mean curvatures at each point on the membrane [51]. For the discretization of elastic energy we follow the method developed by Ramakrishnan *et al.* [52]. The GNR is bound to the membrane through a truncated Lennard-Jones potential (*H*_*LJ*_), and its motion captured by translational and rotational Monte Carlo moves [20].

The MSAD and out of plane motion of the simulated nanoprobe are shown in Fig. 3, for two values of excess membrane area, corresponding to “tense” and “floppy” cases [20, Table S2]. As seen in experiments, the MSAD of the nanorod decreases and becomes more subdiffusive with lower membrane tension [Fig. 3(a)]. Examining the simulated membranes [20, Movie S2, Fig. S11] reveals that the nanorod is transiently caged in the dynamically evolving valleys of the undulating membrane. The same caging effect also causes the $\langle \Delta {\hat{u}}^{2}\rangle$ to be slightly subdiffusive, consistent with experiments. As expected, the out of plane motion of the nanorods on the floppy membrane asymptotes to a higher value [Fig. 3(b)] due to its larger total amplitude of undulations. Moreover, the time-dependent angle *δ* between the simulated membrane normal and that inferred from the nanorod motion in the manner we used in experiments confirms the validity of our approach; they differ by just a few degrees.

The final goal of this study is to demonstrate that our GNR tracking approach is compatible with measurements on the plasma membrane of living cultured cells. GNRs bound to Huh7 cells perform 2D random walks on the membrane and stay in focus for more than 5 min [20, Fig. S13] (unlike rods in the buffer or engulfed by the cell, that rapidly go out of focus). The orientation data resembles rapid diffusive rotation [20, Fig. S14] in a plane tilted by 27 ° with respect to the focal plane. Once the mean normal to the plane, $\bar{n}$, is found, the time-dependent membrane normal vector, $\hat{n}(t)$, shown in Fig. 1(b), can be computed as before. This can then be decomposed into in-plane, Δ*θ*_‖_, and out of plane, Δ*θ*_⊥_, angular displacements, where $\theta_{⊥}=\cos^{-1}\hat{n}\cdot \bar{n}$.

The MSD and a typical trajectory of the GNR at short lag times [Fig. 4(a)] reveal subdiffusive motion of the GNR. The plasma membrane is tethered to underlying actin filaments by integrin proteins which form a structure akin to “picket fences” surrounding “corrals” [Fig. 4(b)] [53–55]. We can interpret the MSD as due to cage diffusion and escape from cages*/*corrals of variable sizes. The MSD crosses over to purely diffusive motion with *D*_*t*_ = 0.06 ± 0.01 *μ*m^2^*/*s at a length scale of ≈(200 nm)^2^, comparable to the largest expected corral size [56].

Both $\langle \Delta \theta_{\|}^{2}\rangle$ and $\langle \Delta \theta_{⊥}^{2}\rangle$ show complex lag-time dependence [Fig. 4(c)]. As in the GUV, the out of plane fluctuation is much slower than the in-plane diffusion of the GNR. At short lag time, *τ* < 10 ms, the *θ*_‖_ shows a pure diffusive behavior, with the diffusivity *D*_*r*_ = 48.0 ± 6.4 rad^2^*/*s comparable with that in a tense pure lipid membrane. At intermediate lag time *τ* > 10 ms, however, the rotational motion is subdiffusive consistent with angular caging.

Due to its coupling to the underlying actomyosin cortex, the undulation dynamics of the plasma membrane on long length and timescales is that of the cortex [57]. Indeed, the subdiffusive exponent of $\langle \Delta \theta_{⊥}^{2}\rangle$ at *τ* > 10 ms resembles the angular motion of cortex-adhered tracers, which report a dynamic shear modulus varying as ~*ω*^0.16^ [58]. At *τ* < 5 ms, $\langle \Delta \theta_{⊥}^{2}\rangle$ increases subdiffusively. We can isolate this short-time motion of the membrane by computing the covariance of the out of plane angle of GNR, *A*(*τ*) = ⟨*θ*_⊥_(*t* + *τ*)*θ*_⊥_(*t*)⟩_*t*_ [Fig. 4(d)], which decays exponentially with a relaxation rate of *ω* = 610 ± 24 s^−1^.

Hypothesizing that the short time dynamics of the normal vector is due to plasma membrane undulations, we can create a simple model by integrating Eq. (1) over a narrow range of wave numbers, *δq*, set by the corral size and the nanorod length, yielding a single exponential decay $A(\tau )=\left(k_{B}T/2\pi \kappa_{c}\right)(\delta q/q)e^{-\omega_{1}\tau }$ consistent with our observations [20]. Using an estimated range for *δq/q* = 0.2−0.9, this provides an estimate of the bending rigidity of the plasma membrane in the range of *κ*_*c*_ ≈ 2−9 *k*_*B*_*T*, at least a factor of 2 smaller than expected [59].

This small inferred bending modulus may be attributed to additional sources of undulation not accounted for by the simple model. For one, the integrins presumably perturb the membrane height, producing a corrugation that leads to dynamic out of plane motion as the nanorod diffuses over it. For another, the presence and diffusion of membrane curvature inducing proteins would also increase the undulation amplitude [60]. Sorting out these multiple contributions will require more extensive future experiments that vary *δq/q* by using different length nanorods and additional modeling.

In conclusion, the nanorod tracking approach we present here enables the reliable measurement of membrane properties of cell-sized GUVs and the undulation dynamics of the plasma membranes of single living cells. Notably, the measurements here are not near their physical limits (e.g., due to photodamage or heating), and so should be readily extendable using faster cameras and brighter laser illumination. This will allow the use of still smaller GNRs and thus the optical measurement of undulations having wavelengths far smaller than the diffraction limit. Since the orientational tracking is polarimetric and can be performed at low magnification, a wide-field camera would also enable high-throughput measurements of different cells or cell regions simultaneously. Last, our approach promises to enable the study of the mechanics of the membranes of procaryotes, subcellular organelles, or the dynamics of cell-cell junctions.

## Supplementary Material

SI Text

Video S1

Video S2

## Figures and Tables

**FIG. 1.**
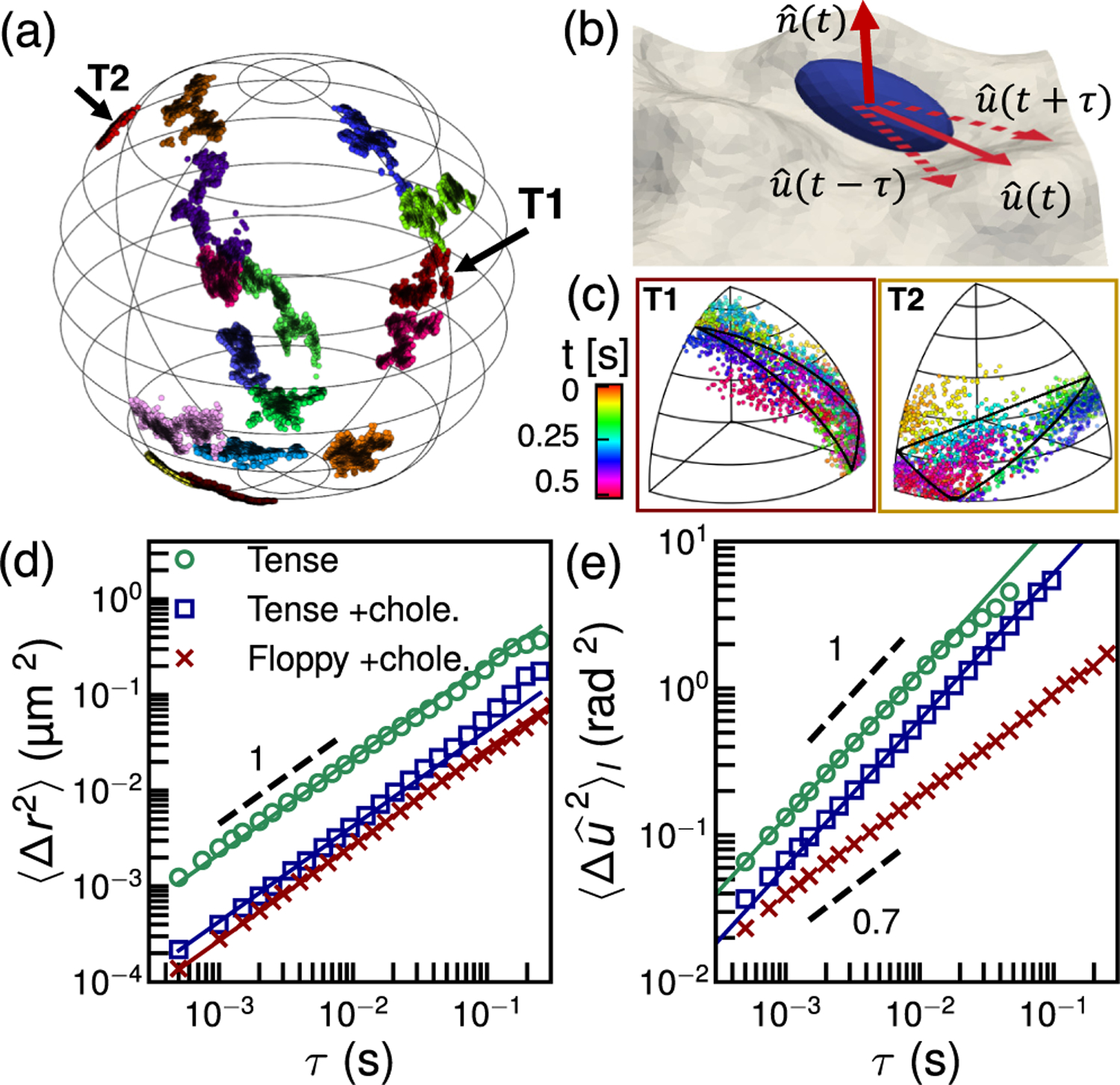
Thermal motion of GNRs on GUVs, varying membrane tension and viscosity. (a) Reconstructed 3D trajectories of the GNR on different parts of a tense (isoosmolar) GUV, each lasting ≈1 s; the GUV radius is *R* = 7 μm. (b) Schematic of a membrane bond nanoprobe, its orientation vector $\hat{u}$, and the membrane normal vector $\hat{n}$. (c) Two typical angular trajectories of GNR orientation, $\hat{u}$, corresponding to the labels T1 and T2 in panel (a), showing rapid in-plane rotation mapped into one octant; loops shown by black lines indicate projection of planes with $\hat{n}={\langle \hat{r}(t)\rangle }_{t}$ into the same octant. (d) MSDs of the GNRs show diffusive translational motion, open symbols, and fits to 4*D*_*t*_*τ*, solid lines. (e) MSADs of the GNRs on the tense GUVs display diffusive motion, on the floppy GUV subdiffusive motion, open symbols, and fits to 4*D*_*r*_*τ*, solid lines. Each MSD is computed using 10 or more GNR trajectories.

**FIG. 2.**
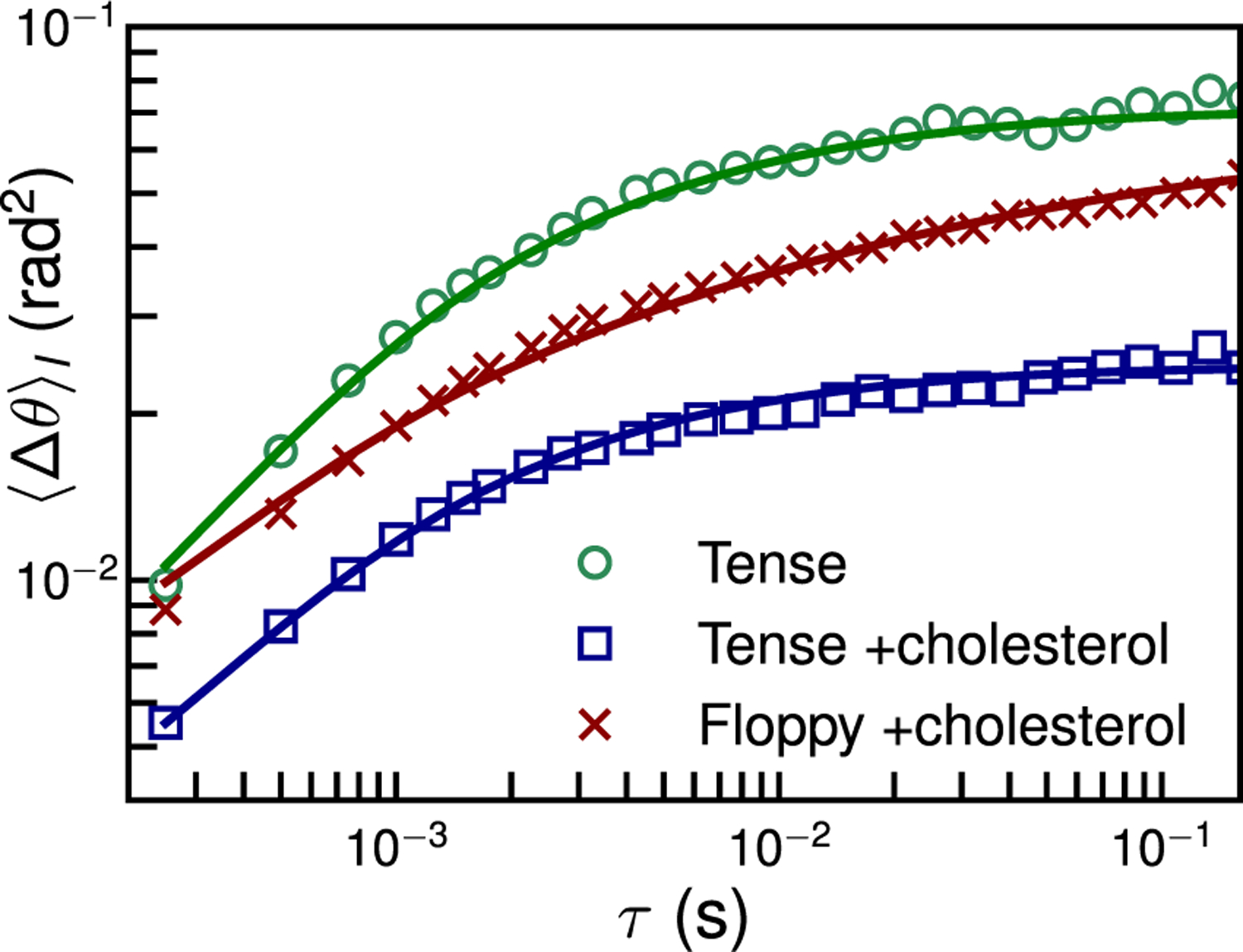
Out of plane angular motion of GNRs on different GUVs reveals the dynamic fluctuations of the membrane normal vector $\hat{n}$. Symbols are the measurement with solid lines fit to Eq. (1).

**FIG. 3.**
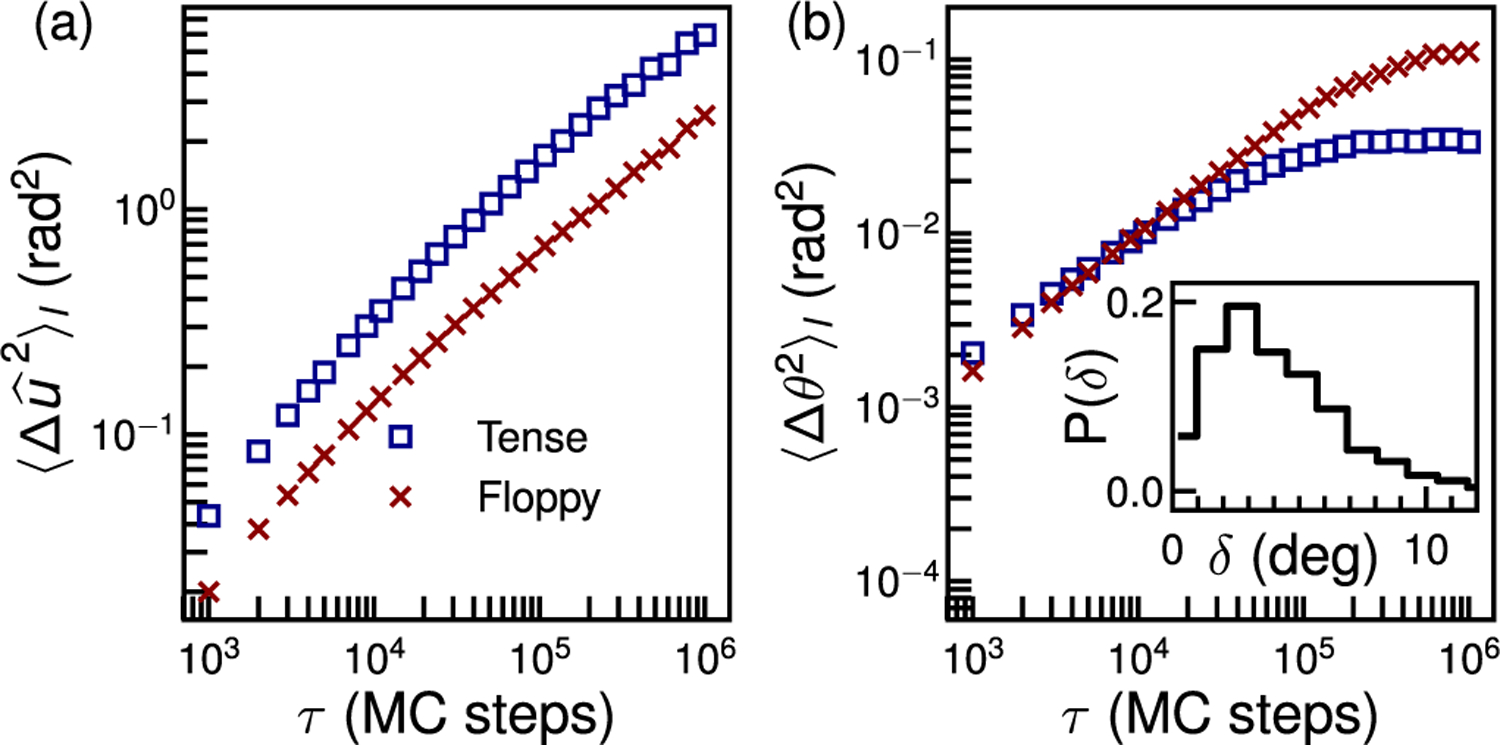
Simulated motion of a nanoprobe on membranes with two different tensions. (a) MSAD of the GNR shows hindrance by membrane undulations, which is greater in the floppy membrane. (b) MSAD of the out of plane motion of the GNR shows larger membrane normal fluctuations on the floppy membrane. Inset: histogram of the angular difference between the simulated membrane normal $\hat{n}$ and that reported by the nanoprobe.

**FIG. 4.**
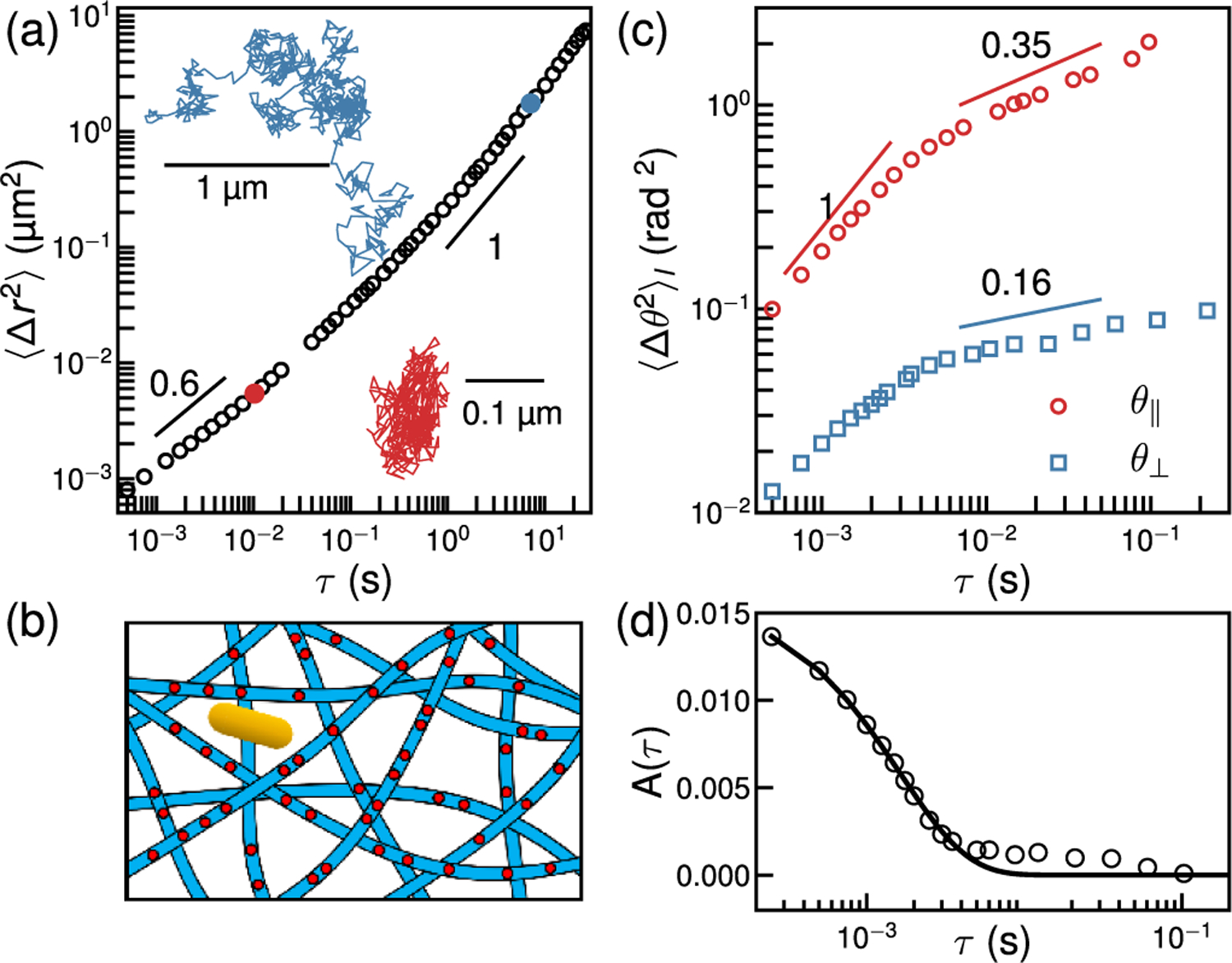
GNR tracking on a cell plasma membrane reveals translational caging and rapid normal vector fluctuations. (a) MSD of the GNR shows subdiffusive caging at short lag times, and diffusion at long lag times, illustrated by the red and blue trajectories spanning 10 ms and 7 s, respectively. Eye guides show the logarithmic slope. (b) Schematic of a GNR caged by integrin proteins (red circles), anchored to actin filaments under the membrane. (c) MSAD of the in-plane and out of plane motion of the GNR show rapid motion at short times and caging at long lag times resembling cell cortical fluctuations. (d) The covariance of the out of plane angle reveals the rapid decay of plasma membrane normal fluctuations relative to the cortex.
