# Supplementary material for: Probing lipid membrane bending mechanics using gold nanorod tracking
Source: Phys Rev Res. Author manuscript; Available in PMC 2022 Apr 1. (PMC8975244; doi:10.1103/physrevresearch.4.l012027)
Supplement: SI Text [file NIHMS1786796-supplement-SI_Text.pdf]

Supplementary Material for
Probing lipid membrane bending mechanics using gold nanorod tracking

Mehdi Molaei and John C. Crocker
Chemical and Biomolecular Engineering, University of Pennsylvania, Philadelphia, PA 19104

Sreeja Kutti Kandy and Ravi Radhakrishnan
Bioengineering, University of Pennsylvania, Philadelphia, PA 19104

Zachary T. Graber and Tobias Baumgart
Chemistry, University of Pennsylvania, Philadelphia, PA 19104

S1. EXPERIMENTAL METHODS

A. Sample Preparation

GNR solution: The carboxyl functionalized GNR solution was purchased from Nanopartz (A12-40-850-TC) with GNR concentration of 1.1×10^{12} nps/mL. In a 1.5 mL microcentrifuge vial, we mixed 20 µL of this GNR solution with 0.98 mL PBS buffer with pH of 7.4 to make a dilute GNR suspension. Magainin 2 peptide was purchased from Genscripts (RP11232-0.5). 1 mL DI water was added to 10 mg magainin 2 to make a 4 mmol peptide solution. To functionalize GNRs with the peptide molecules, we used a protocol designed to conjugate nanoparticles with nuclear localization signal (NLS) peptide [1] with minor alterations. 1 ml DI water was added to 0.19 mg of N-ethyl-N'-dimethylaminopropyl-carbodiimide (EDC, from Sigma-Aldrich E6383-1G) to make 1 mmol EDC solution. In a separate vial, 1 mL DI water was added to 0.11 mg of N-Hydroxysuccinimide (NHS, from Sigma-Aldrich 130672-5G) to yield 1 mmol NHS solution. After preparing these samples, we added 10 µL of the EDC solution and 5 µL of the NHS solution to the dilute GNR suspension and vortexed for 10 seconds. Then, we added 20 µL of the peptide solution to the sample. The sample was stirred for 48 hours. At the end, the sample was centrifuged at 1000 rcf and resuspended in the PBS buffer twice to remove the excess peptides. To verify that the GNRs were not aggregated throughout the process, the uv-vis absorbance of the final sample was measured and compared with the absorbance of the original sample.

GUV solution: Giant Unilamellar Vesicles (GUVs) were prepared from lipid stocks of 1,2-dioleoyl-sn-glycero-3-phosphocholine (DOPC), 1,2-dioleoyl-sn-glycero-3-phospho-L-serine (DOPS), and cholesterol using the standard method of electroformation [2, 3]. Briefly, the lipid stocks were prepared in organic solvent with the desired lipid composition and spread on indium tin oxide (ITO) coated slides. To remove all traces of organic solvent and form a dry lipid film, the lipid-coated slides were placed under vacuum for two hours. An electroformation chamber was formed using two lipid-coated ITO slides with 0.8 mm rubber spacers. Inside the electroformation chamber the lipid film was hydrated with 400-450 µL of 0.3 M sucrose. Electroformation was performed by applying a 10 Hz, 2-4 Vpp electric field over two hours to form the final GUV dispersion. To perform the experiment, we add 10 µL of the peptide coated GNR solution to 200 µL of the GUV solution. The mixed solution was loaded between two #1.5 coverslips and is sealed carefully with vacuum grease.

Huh7 cells: Huh7 cells were cultured following the standard protocol. Cells were grown in a 37 °C incubator under 5% CO2. They are cultured in 35 mm dish with #1.5 coverslip in the middle (Mattek, P35GCOL-1.5-10-C) in 2 mL of DMEM with 10% FBS and penicillin/streptomycin. Cells were grown to ∼50% confluence which took about 2 days. Before imaging, the culture medium were replaced with a PBS buffer with pH 7.3, and 10 µL GNR solution was added.

B. Imaging and particle tracking

Highly polarized light scattered from GNRs are imaged using a custom-built laser-illuminated dark field microscope. The detail of the microscope is provided in an earlier publication [4]. The single longitudinal mode diode pumped solid state laser $\lambda = 670$ nm; $P = 300$ mW (Shanghai dream lase, SDL-671-30) provides an illumination intensity of roughly $200\,\mu W/\mu m^2$ at the GNR. We collect the back-scattered light using a 100X oil-immersion objective (Leica HCX PL APO 100X-1.4) and a high speed CMOS Phantom IV camera (Vision Research) to collect and simultaneously image the light in two orthogonally polarized channels with effective pixel size of 0.1 µm. All imaging was performed at room temperature.

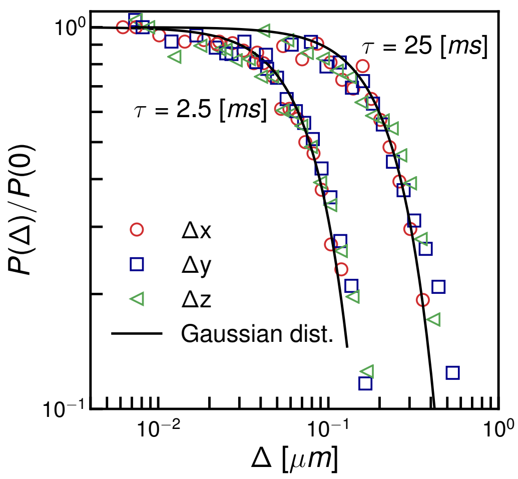

FIG. S1. The distribution of displacements in all three direction, or van Hove functions for $\tau = 2.5\,\mathrm{ms}$ and $\tau = 25\,\mathrm{ms}$, follow normal distributions.

The lateral motion, xy, of the GNR is tracked using the centroid of the DFM images. The center x_c, y_c and radius, R, of the GUV are determined by the phase contrast microscopy. The position of GNR in z direction is then computed through

$$z = [R^2 - (x - x_c)^2 - (y - y_c)^2]^{\frac{1}{2}}. \tag{S1}$$

The probability of the GNR displacement in lateral and in-depth direction Fig. S1 shows isotropic diffusion process with Gaussian distributions insuring the accuracy of 3-d trajectories. The MSD of a particle diffusing over the surface of a sphere follows

$$\langle \Delta \boldsymbol{r}^2(\tau) \rangle = 2R^2(1 - \exp[-\frac{2D_t\tau}{R^2}]), \tag{S2}$$

where $D_t = k_BT/\gamma_t$ is the translational diffusion coefficient, and γ_t, k_B, and T are the translational drag coefficient, Boltzmann constant, and the temperature. For $\tau \ll D_t/R^2$, Eqn. (S2) simply becomes a Brownian random walk on a 2D plane with $\langle \Delta \boldsymbol{r}^2(\tau) \rangle = 4D_t\tau$.

S2. MAPPING BOUNDED MSADS TO UNBOUNDED MSADS

Consider a rod anchored on a 2D plane with normal vector of

$$\hat{n} = \sin\beta\cos\phi\hat{e}_x + \sin\beta\sin\phi\hat{e}_y + \cos\beta\hat{e}_z, \tag{S3}$$

where ϕ and β are the azimuthal and polar angles. Now let the rod rotationally diffuse in this 2D plane with diffusion coefficient of D_r. The unit vector of the rod mapped on the plane is $u' = \cos\theta\hat{e}'_x + \sin\theta\hat{e}'_y$ where $\hat{e}'_{x,y}$ are the unit vectors of the coordinate system aligned on the 2D plane and θ is generated by random rotational Brownian motion where $\langle \Delta\theta^2(t) \rangle = 2D_rt$. It can be shown that the unit vector of the rod is

$$\hat{\boldsymbol{u}} = [\cos\theta - (1-\cos\beta)\cos\phi\cos(\theta-\phi)]\hat{e}_x + [\sin\theta - (1-\cos\beta)\sin\phi\sin(\theta-\phi)]\hat{e}_y - \sin\beta\cos(\theta-\phi)]\hat{e}_z, \tag{S4}$$

which follows a circular loop. For instance, if the rod diffuses on a plane with $\beta = 30°$ and $\phi = 45°$, the unit vector of the rod will follow the circular path shown in Fig. S2(a).

If we were measuring the orientation of this rod with our imaging technique, because $\hat{\boldsymbol{u}}$ is mapped to an octant of a sphere, the measured orientation vector would have followed a path shown in Fig. S2(b). Video 1 respectively show the random motion of the the rod diffusing on this plane and its mapped orientation to the octant. The MSAD of the fully resolved unit vector is an exponential function, $\Delta\hat{\boldsymbol{u}}^2(\tau) = 2(1 - \exp[-D_rt])$, and MSAD of the measured unit vector follows an stretched exponential function

$$\langle \Delta\hat{\boldsymbol{u}}(\tau)^2 \rangle = \Delta\hat{\boldsymbol{u}}^2_\infty(1 - \exp[-(kD_r\tau)^\xi]), \tag{S5}$$

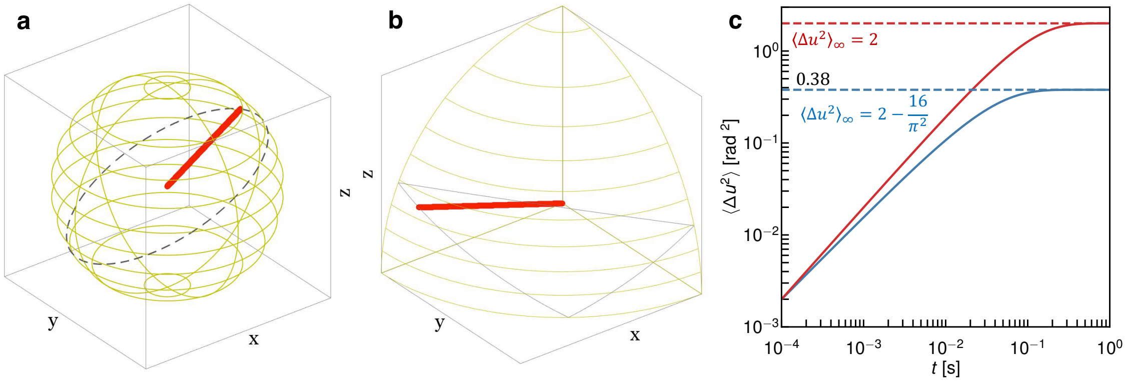

FIG. S2. Motion of rod on a tilted plane with $\beta = 30^\circ$ and $\phi = 45^\circ$. a) $\hat{\boldsymbol{u}}$, the unit vector pointing the major axis of the rod, red thick line, follows a circular path shown with gray dashed lines. b) Mapped orientation vector, $|\hat{\boldsymbol{u}}|$ follows a peculiar loop in octant of sphere shown by grey line. c) MSAD of the unite vector $\hat{\boldsymbol{u}}$ red line, and its mapped on the octant, $|\hat{\boldsymbol{u}}|$ blue line follow exponential and stretched exponential functions respectively.

as shown in Fig. S2(c), where $k = 4.04$ and $\xi = 0.93$ are the parameters of the stretched exponential, and $\Delta\hat{\boldsymbol{u}}^2_\infty$ is the asymptote value. For a point moving randomly on a circle, the asymptote value of the MSD is calculated as

$$\Delta\hat{\boldsymbol{u}}^2_\infty = \frac{1}{4\pi^2}\int_0^{2\pi}\int_0^{2\pi}[1-\cos(x_1-x_2)]dx_1dx_2 = 2. \qquad \text{(S6)}$$

For a point moving moving on an arc of size $\pi/2$, the asymptote is calculated as

$$\Delta\hat{\boldsymbol{u}}^2_\infty = \frac{1}{4\pi^2}\int_0^{\pi/2}\int_0^{\pi/2}[1-\cos(x_1-x_2)]dx_1dx_2 = 2 - \frac{16}{\pi^2}. \qquad \text{(S7)}$$

Simulation of the rod on planes with any polar and azimuthal angles shows that the MSAD of the unit vector does not depend on the orientation of the plane.

Importantly, we can compute a lag-time independent mapping between the measured MSAD bounded to an octant and a physical unbounded MSAD that increases linearly as the rod orientation tumbles end over end, by inverting Eq. S5:

$$\langle\Delta\hat{\boldsymbol{u}}^2(\tau)\rangle_l = \frac{4}{k}\left[-\ln\left(1-\frac{\langle\Delta\hat{\boldsymbol{u}}^2(\tau)\rangle}{\Delta\hat{\boldsymbol{u}}^2_\infty}\right)\right]^{\frac{1}{\xi}}, \qquad \text{(S8)}$$

as shown previously [4]. Measured bounded MSADs of the GNRs on tense and floppy GUVs and their corresponding scaled unbounded MSADs are shown in Fig. S3. The unbounded MSADs are reported in the main text, Fig. 1(e).

In the main text, we have associated $\langle\Delta\hat{\boldsymbol{u}}^2(\tau)\rangle$ solely with the in-plane rotational motion of the GNR, but one could argue that the translational motion of a GNR on the GUV can also contribute to the change in the orientation vector. However, since $D_t/R^2 \ll D_r$ we can confidently neglect such contribution. Nevertheless, we have generated a random rotational motion of a GNR on a GUV with and without random walk. The result shown in Fig. S4 confirms our assessment. In a similar way, it can be shown that out of plane undulation of the membrane is not large enough to contribute to $\langle\Delta\hat{\boldsymbol{u}}^2(\tau)\rangle$.

S3. ESTIMATING MEMBRANE VISCOSITY AND EFFECTIVE NANOROD LENGTH

The drag coefficient of the GNR depends on the 2D membrane viscosity, η_m, bulk fluid viscosity η, and the geometry and orientation of the rod. In the limit that $a < l \ll \eta_m/\eta$, the drag coefficient of the rod become independent of the rod orientation and aspect ratio [5], and Saffmann and Delbrük (SD) model for a disklike membrane inclusion with the effective diameter of l_{eff} can be used to estimate rotational and translation drag coefficients [6].

$$\gamma_t = 2\pi\eta l_{eff}[\frac{\rho}{\ln\rho - \gamma_E}], \qquad \text{(S9)}$$

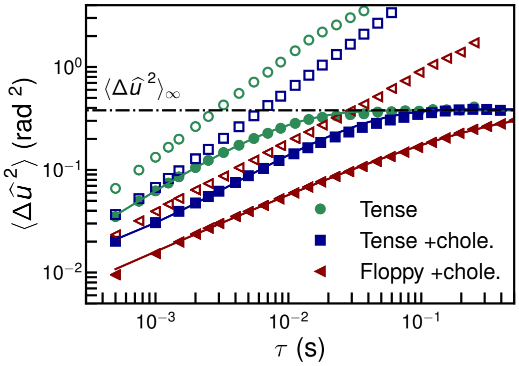

FIG. S3. Measured bounded MSADs, closed symbols, and scaled unbounded MSADs, open symbols, of the GNRs on GUVs. Lines are fits to Eqn. (S5).

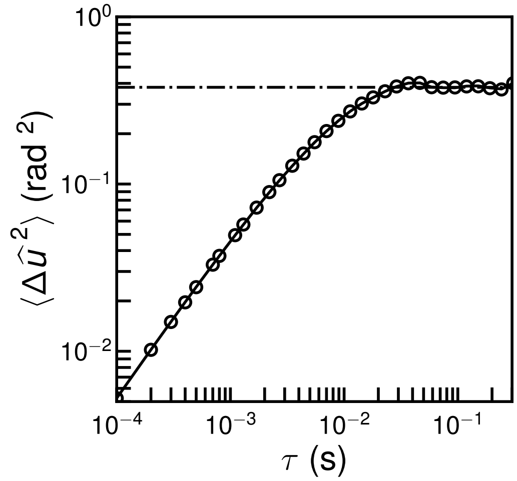

FIG. S4. MSAD of random motion of a nanorod with in-plane rotational diffusion of $D_r = 27\,\mathrm{rad}^2/\mathrm{s}$ and translational diffusion of $D_t = 1\,\mathrm{\mu m}^2/\mathrm{s}$, symbols, and $D_t = 0$, solid line. The GUV radius is $R = 7\,\mathrm{\mu m}$, similar to the experiment.

$$\gamma_r = \frac{\pi \eta l_{eff}^3}{2}\rho, \tag{S10}$$

where, γ_E is Euler-Mascheroni constant, $\rho = 2\eta_m/l_{eff}\eta$ is the dimensionless length indicating the importance of membrane mechanics to bulk hydrodynamic. For an object diffusing on a viscous membrane, the physical size of the probe often is not considered as the effective size for diffusion process; in this setting, the number of bounding sites to the bilayer and also distortion induced by the object determined the effective size [7]. Hence, we consider the effective length of the GNR as an unknown parameter in the diffusion process. Knowing both rotational and translational drag coefficients of the GNR, however, we determine both membrane viscosities and effective size of the GNRs. Applying SD model, we determine the relation between the ratio of translational to rotational drag coefficients, $\alpha = \gamma_t/\gamma_r^{1/3}(4\pi\eta)^{2/3}$ and length scale associated with the membrane viscosity and the bulk fluid viscosity.

$$\alpha = \frac{\rho^{2/3}}{ln\rho - \gamma_E} \tag{S11}$$

For example, for the tense GUV with no cholesterol case, $\alpha = 2.73 \pm 0.3$ yielding $\rho = 11.4 \pm 0.1$; placing this value in Eqn. (S10) the effective length of the GNR is determined, $l_{eff} = 202 \pm 25\,\mathrm{nm}$. The membrane viscosity is simply calculated, $\eta_m = \rho\eta l_e ff/2 = 1.2 \pm 0.1\,\mathrm{nPa}$. The membrane viscosity in all the experiments done here is large enough to justify using the SD model, effectively treating GNRs as disks with diameters equal to l_{eff}.

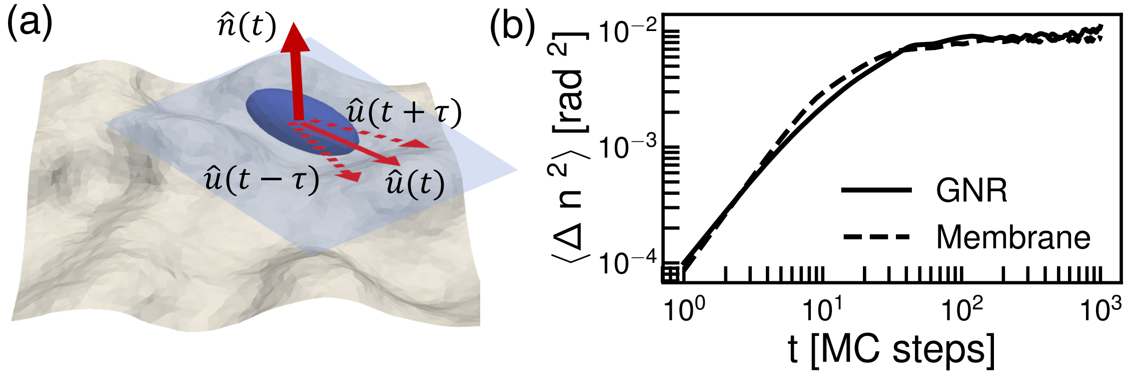

FIG. S5. a) Measurement of membrane normal vector from orientation of membrane bond GNR. b) MSAD of the membrane normal vector, $\hat{n}_s$ dashed line, and estimated normal vector, $\hat{n}$ solid line

S4. ESTIMATING MEMBRANE NORMALS FROM DYNAMIC GNR ORIENTATION

In this paper, we infer the fluctuating normal of a lipid membrane from the dynamic orientation of a nanorod lying flat on the membrane and undergoing rapid rotational diffusion within the plane of the membrane. To estimate the angular error of this procedure, we use the simulated motion of a nanorod on membrane simulated using a Monte Carlo approach, described below in Section S7. First, we compute the local, time-averaged normal vector of the segment of the membrane associated with the rod, $\hat{\boldsymbol{n}}_s(t)$. We then estimate the normal vector using the same analysis method used for the experimental data, namely fitting a plane to a few consecutive time points of the nanorod orientation vector, as sketched in Fig. S5(a). The angular difference between the membrane normal vector and the estimated normal $\delta = cos^{-1}(\hat{\boldsymbol{n}}_s \cdot \hat{\boldsymbol{n}})$ indicates the agreement of the estimated values with normal vector, shown in the inset to Fig. 3(b). Moreover, the MSAD of the membrane fluctuation can be estimated by measuring MSAD of the normal vector of GNR, $\hat{\boldsymbol{n}}$, as plotted in Fig. S5(b).

A separate issue related to the effect of mapping the nanorod orientations to an octant by our experimental method. A simple numerical check confirms that rescaling the MSAD removes any bias this might cause. Experimentally, the out of plane angular motion of the GNR is determined by tracking the angle between $\hat{\boldsymbol{n}}$ and the average normal vector of the plasma membrane $\theta_\perp = \cos^{-1} \bar{\boldsymbol{n}} \cdot \hat{\boldsymbol{n}}$ or the angle between $\hat{\boldsymbol{n}}$ and the position vector of the GNRs on the GUVs $\theta_\perp = \cos^{-1} \hat{\boldsymbol{r}} \cdot \hat{\boldsymbol{n}}$. This process bounds all the angle information to $0 < \theta_\perp < \pi/2$. We then apply our previously developed approach that computes a lag-time independent mapping between bounded and unbounded MSADs [4]. To ensure that this mapping approach faithfully maps bounded $\langle \Delta\theta^2 \rangle$ to unbounded ones $\langle \Delta\theta^2 \rangle_l$, we simulate a random angular walk of the normal vector. Without loss of generality, we set $\bar{\boldsymbol{n}} = \hat{\boldsymbol{e}}_z$ and generate two sets of random walks for the angular motion of $\theta_{zx} = \tan^{-1}(n_x/n_z)$ and $\theta_{zy} = \tan^{-1}(n_y/n_z)$. Both of these walks are generated in a way that their MSDs follow Kelvin-Voigt model [8] which has qualitatively similar time dependence to the measured $\langle \Delta\theta_\perp^2 \rangle$. Figure S6 shows the MSAD of this randomly generated walk, $\langle \Delta\theta_{xz}^2(\tau) \rangle + \langle \Delta\theta_{yz}^2(\tau) \rangle$. The normal vector of the membrane is constructed as

$$\hat{\boldsymbol{n}} = \frac{1}{(\tan^2\theta_{xz} + \tan^2\theta_{xy} + 1)^{\frac{1}{2}}} [\tan\theta_{xz}\hat{\boldsymbol{e}}_x + \tan\theta_{yz}\hat{\boldsymbol{e}}_y + \hat{\boldsymbol{e}}_z], \tag{S12}$$

and $\theta_\perp = \cos^{-1}\left(\left[\tan^2\theta_{xz} + \tan^2\theta_{xy} + 1\right]^{-1/2}\right)$. The MSAD of this out of plane angle, $\langle \Delta\theta_\perp^2 \rangle$ is shown in Fig. S6. The mapped MSAD is then obtained from

$$\langle \Delta\theta_\perp^2(\tau) \rangle_l = \frac{\pi}{0.85} \left[-\langle \Delta\theta^2(\infty) \rangle \ln\left(1 - \frac{\langle \Delta\theta_\perp^2(\tau) \rangle}{\langle \Delta\theta^2(\infty) \rangle}\right) \right]^{\frac{1}{0.95}}, \tag{S13}$$

where $\langle \Delta\theta^2(\infty) \rangle = \pi^2/24$. Figure S6 shows that that $\langle \Delta\theta_\perp^2(\tau) \rangle_l$ closely follows original MSAD, $\langle \Delta\theta_{xz}^2(\tau) \rangle + \langle \Delta\theta_{yz}^2(\tau) \rangle$.

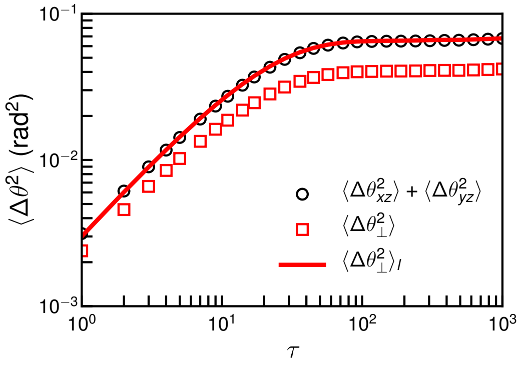

FIG. S6. MSAD of the out of plane motion of a membrane with a normal vector with a random walk following Kelvin-Voigt model. The logic behind selecting this model is that the measured out of plane MSADs, Fig. 2, follow a similar MSD of colloids in a Kelvin-Voigt materials.

S5. RELATING GNR MOTION TO THE SEIFERT-LANGER HYBRID MODEL OF MEMBRANE FLUCTUATION

In this section, we use previously developed models for relaxation dynamics of membrane fluctuations to establish a model for the out-of-plane orientational fluctuations of membrane bound nanorods. The relaxation dynamics of lipid membranes are determined by their elastic and drag forces. In a classical picture by Helfrich [9], for small wave numbers and length scales far larger than their thickness, a lipid bilayer system is treated as a 2D incompressible sheet with small surface tension and bending modulus. In this picture, a membrane can undergo shape change with no longitudinal deformation. Hence, surface tension along with bending stiffness and bulk fluid viscosity exclusively govern curvature fluctuations of the membrane. This approach was extended by a continuum model developed by Seifert and Langer (SL) [10] that considers the bilayer structure over a wider range of length scales and includes additional elastic and dissipative forces. In the SL model, Unlike Helfrich', a local curvature change in the length scale of membrane thickness can generate longitudinal deformations in each monolayer. Therefore, in addition to the bending of the membrane, stretching of the monolayers can also generate elastic forces, and in addition to the viscosity of the bulk fluids and the membrane viscosity, the frictional coupling between the two monolayers also generates dissipative forces.

The SL model begins with the height-height fluctuation of a planar membrane [10]:

$$\left\langle h_q(t+\tau)h_q^*(t)\right\rangle_t = \frac{k_BT}{\kappa_c q^4 + \sigma q^2}\left[A_1(q)e^{-\omega_1(q)\tau} + A_2(q)e^{-\omega_2(q)\tau}\right], \tag{S14}$$

where the respective amplitude $A_1(q)$ and $A_2(q) = 1 - A_1(q)$, and the relaxation rates, $\omega_{1,2}$, can be explicitly determined in specific regimes separated by the crossover wavenumbers [11–14]. For the wavenumbers below $q_1 \equiv 2\eta\varepsilon/b\tilde{\kappa}_c$ fluctuation of the membrane is damped by viscous forces within the bulk fluids. For the wavenumbers above $q_2 \equiv \sqrt{2b/\eta_m}$, the main dissipation source is the membrane viscosity rather than slipping of the monolayers. On intermediate wavenumbers $q_1 < q < q_2$, the bending mode relaxes with the rate of $\omega_2 = \tilde{\kappa}_c q^3/4\eta$, and slipping mode relaxes with $\omega_1 = \varepsilon\kappa_c q^2/2\tilde{\kappa}_c b$. In this regime, $A_1 = 2\varepsilon d^2/\tilde{\kappa}_c = 1 - \kappa_/\tilde{\kappa}_c$, and Eqn. (S14) can be simplified as

$$\left\langle h_q(t+\tau)h_{-q}(t)\right\rangle_t = \frac{k_BT}{\kappa_c q^4 + \sigma q^2}\left[(1 - \frac{\kappa_c}{\tilde{\kappa}_c})e^{-\omega_1(q)\tau} + \frac{\kappa_c}{\tilde{\kappa}_c}e^{-\omega_2(q)\tau}\right]. \tag{S15}$$

We now link membrane height fluctuation to orientational fluctuation of the nanorods. first, considering that the size of the nanorods are orders of magnitude smaller than the size of the GUVs, we assume the membrane shape is nearly flat $z = h(x, y)$. The membrane normal then is determined by the gradient of the surface function, $n = \nabla\Phi$, where Φ has a form of $\Phi = z - h(x, y)$. The local out of plane angle of the membrane is $\cos^2\theta = 1/(n_x^2 + n_y^2 + 1)$. Since $n_x = \partial h/\partial x$ and $n_y = \partial h/\partial y$, we can write $\tan^2\theta = (\partial h/\partial x)^2 + (\partial h/\partial y)^2$, and considering the paraxial approximation, $\theta^2 \approx (\partial h/\partial x)^2 + (\partial h/\partial y)^2$. In a Fourier space, one can get $\theta_q^2 = q_x^2 h_{xq}^2 + q_y^2 h_{yq}^2$, where $q_{x,y}$ are the wave numbers in x and y directions. Finally, applying binomial approximation, we estimate out of plane angle of the membrane based on its local height in a Fourier space, $\theta_q \approx q h_q$.Using this approximation in Eqn. (S15) leads to

$$\left\langle \theta_q(t+\tau)\theta_{-q}(t)\right\rangle_t = \frac{k_BT}{\kappa_c q^2 + \sigma}\left[(1 - \frac{\kappa_c}{\tilde{\kappa}_c})e^{-\omega_1(q)\tau} + \frac{\kappa_c}{\tilde{\kappa}_c}e^{-\omega_2(q)\tau}\right]. \tag{S16}$$

The mean square displacement of the out of plane angle then can be calculated from the angle correlation function by summing over the effective wave numbers

$$\begin{aligned}\Delta\theta^2(\tau) &= \langle\theta(t+\tau)-\theta(t)\rangle \\ &= \frac{1}{4\pi^2}\int_{q_{min}}^{q_{max}}\Big[2\langle\theta_q(t)\theta_{-q}(t)\rangle - 2\langle\theta_q(t+\tau)\theta_{-q}(t)\rangle\Big]2\pi q dq.\end{aligned} \tag{S17}$$

Where we considered $\langle\theta_q(t)\theta_{-q}(t)\rangle$ to be constant over time as ergodicity requires. Using Eqn. (S16) and $\langle\theta_q\theta_{-q}\rangle = k_BT/(\kappa_c q^2+\sigma)$, we immediately obtain

$$\langle\Delta\theta^2(\tau)\rangle = \frac{k_BT}{\pi}\int_{q_{min}}^{q_{max}}\frac{qdq}{\kappa_c q^2+\sigma}\left[1-\left(1-\frac{\kappa_c}{\tilde{\kappa}_c}\right)e^{-\omega_1\tau}-\frac{\kappa_c}{\tilde{\kappa}_c}e^{-\omega_2\tau}\right]. \tag{S18}$$

The integral is computed numerically using global adaptive quadrature method.

Furthermore, we evaluate the covariance of the out of plane angle of the nanorod, $A(\tau) = \langle\theta_\perp(t+\tau)\theta_\perp(t)\rangle_t$, when it is only effected by the narrow range of undulation modes with wave number of $q'-\delta q'/2 < q < q'+\delta q'/2$. We start by integrating Eqn. S16 over the narrow range of q,

$$A(\tau) = \frac{k_BT}{2\pi}\int_{q'-\delta q'/2}^{q'+\delta q'/2}\frac{qdq}{\kappa_c q^2+\sigma}\Big[(1-\frac{\kappa_c}{\tilde{\kappa}_c})e^{-\omega_1(q)\tau}+\frac{\kappa_c}{\tilde{\kappa}_c}e^{-\omega_2(q)\tau}\Big], \tag{S19}$$

and simply use trapezoidal rule to approximate this integral

$$A(\tau) = \frac{k_BT}{2\pi}\frac{q'\delta q'}{\kappa_c q'^2+\sigma}\Big[(1-\frac{\kappa_c}{\tilde{\kappa}_c})e^{-\omega_1(q')\tau}+\frac{\kappa_c}{\tilde{\kappa}_c}e^{-\omega_2(q')\tau}\Big], \tag{S20}$$

Since the narrow range of q includes the wave number set by the size of the GNR, we expect that $\sigma \ll \kappa_c q'^2$ and $\kappa_c \ll \tilde{\kappa}_c$ which simplifies above equation as

$$A(\tau) = \frac{k_BT}{2\pi\kappa_c}\frac{\delta q}{q}e^{-\omega_1(q')\tau}. \tag{S21}$$

We assume that the lower range of the undulation wave number is set by the typical distance between pinning sites of the membrane to the cell cortex. While this value varies in different cells it is expected to be close to the typical range of the F-actin mesh sizes of the cell cortex, ($l_{pin} \approx 30-300$ nm) [15, 16]. The upper range of the undulation wave number is set by the size of the GNR. Hence, we estimate that $\delta q/q = 2(q_{max}-q_{min})/(q_{max}+q_{min}) \approx 2(l_{pin}-l)/(l_{pin}+l)$.

S6. SENSITIVITY AND REPEATABILITY ANALYSIS

First to verify that our numerical fitting of Eqn. S18 to the experimental data is reliable, we perform a χ^2 analysis at logarithmically spaced values of the relevant parameters κ_c, b, and σ . Specifically, χ^2 is calculated by comparison of measured and computed $\Delta\theta^2(\tau)$ using:

$$\chi^2(\kappa,\ b,\ \sigma) = \sum_\tau \frac{\left[\Delta\theta^2(\tau)_{\text{exp}} - \Delta\theta^2(\tau)|_{\kappa,b,\sigma}\right]^2}{\Delta\theta^2(\tau)|_{\kappa,b,\sigma}}. \tag{S22}$$

Figure S7 shows that the χ^2 is smallest at the values obtained from the fitting increases rapidly as κ_c, b, and σ deviate from the fitted values, and does not suggest any significant correlation between the three parameters.

We also evaluate the sensitivity of the measurement to nanorod length polydispersity, which affects the largest wave number of undulations sampled by the GNR, q_{max}. While the minimum wave number, q_{min}, (set by the diameter of a GUV) does not have a pronounced impact on the fitting values, the fit values of the tension and intermonolayer friction coefficient are quite sensitive to q_{max}, Fig. S8. Therefore, we fit the measurement using the range of q_{max} determined by $2\pi/(l \pm l_s)$, where l_s is the standard deviation of GNR length, which contributes significantly to the uncertainty of those two parameters, relative to the noise in the measurements. A similar approach yields estimates of $\delta q/q$ in the range $0.2-0.9$.

To assess the repeatability of the measurement results (i.e. sensitivity to unknown sources of measurement error), we performed thrree replicates of the measurement of lipid membrane mechanics for tense GUVs with cholesterol. Figure S9 shows the out of plane motion of GNRs on three different replicates with the fit to Eqn. 1 and Eqn.2.

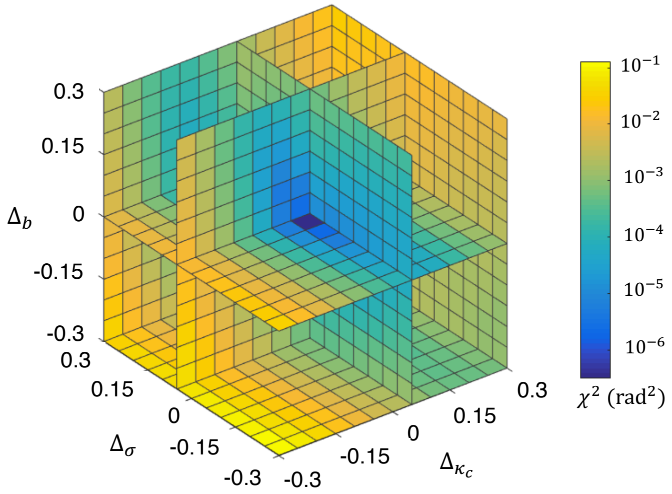

FIG. S7. χ^2 test to evaluate sensitivity of the fitting for out of plane motion of GNR on the isoosmolar GUV with no cholesterol. χ^2 is calculated using Eqn. S18 and Eqn. S22 at $\kappa = \kappa_{fit} + \Delta\kappa$, $b = b_{fit} + \Delta b$, and $\sigma = \sigma_{fit} + \Delta\sigma$, where subscription 'fit' indicates the values obtained from the fitting.

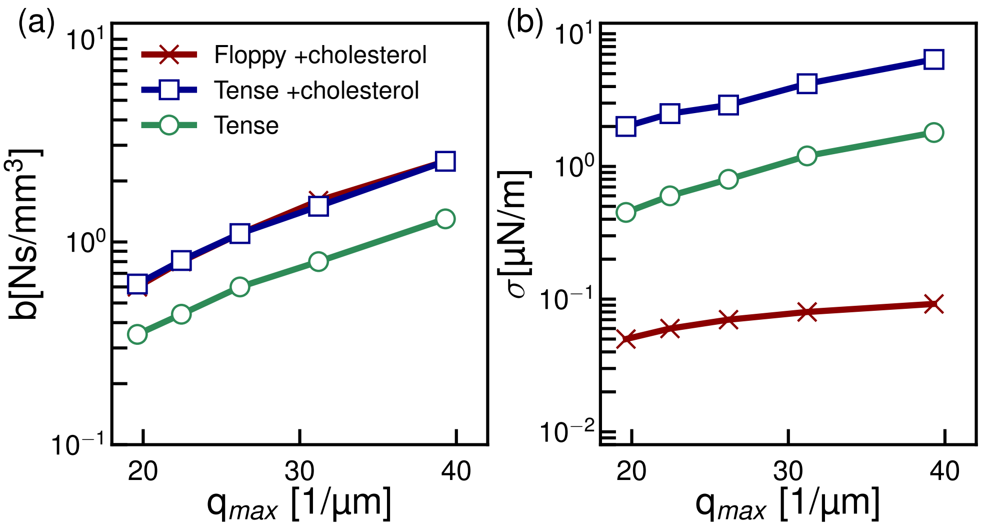

FIG. S8. Fitting sensitivity of intermonolayer friction coefficient, b, and tension, σ, to the uncertainty in the value of q_{max}. The fitting value of κ_c, not shown here, does not have strong sensitivity to the value of q_{max}. Tense membranes are show higher sensitivity of inferred tension on q_{max} than floppy ones.

The result of the fitting is summarized in Table S1 along with the the membrane viscosity and effective length of the GNRs estimated from the in-plane translational and rotational motion of the GNRs as described in Sec. S3. The large variation in the effective size of the GNRs in three samples which may be due to a variable number of links between the lipid layers and GNRs [7].

S7. MEMBRANE-GNR MONTE CARLO MODEL

Dynamically triangulated Monte Carlo model for membrane: We consider a patch of fluctuating membrane bounded by a square frame of size L with periodic boundary conditions in lateral directions. The continuum surface of the membrane is discretized into N_t triangles which connects N_v vertex points through N_l links [17]. The membrane

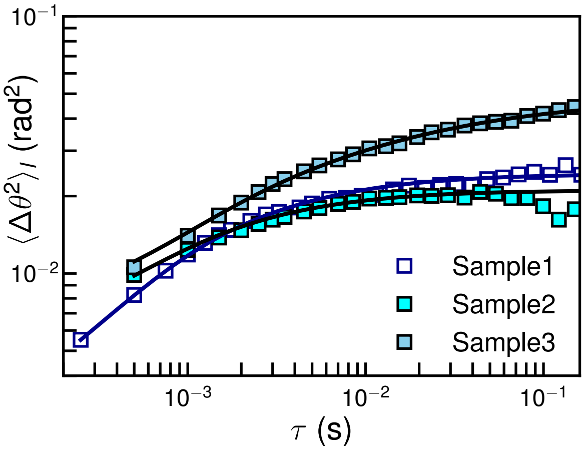

FIG. S9. Out of plane angular motion of GNRs on three replicates of tense GUVs with cholesterol. Symbols are the measurement with solid lines fit to Eqn.1 and Eqn. 2. Sample I is reported in the main text.

Sample	$\eta_m(nPasm)$	$\kappa_c(k_B\ T)$	$b(Pa\ s\ nm^{-1})$	$\sigma(\mu N\ m^{-1})$	$l_{eff}\ (nm)$
I	17.7	19.2	1.2	4.5	67
II	18.1	19.4	1.5	8.5	152
III	18.6	18.7	2.7	0.4	196

TABLE S1. Summary of the lipid membrane properties of the tense GUVs with cholesterol. Result of sample I is reported in the main text.

conformations are governed by elastic energy of the surface given by Canham-Helfrich approximation for bilayer membranes [9]; the discretized form of hamiltonian is given by

$$\mathbf{H}_{\text{elastic}} = \frac{\kappa_c}{2}\sum_{v=1}^{N_v}(c_{1,v} + c_{2,v})^2 A_v. \tag{S23}$$

Where, κ_c is the bending rigidity of membrane, A_v is the area and $c_{1,v}$ and $c_{2,v}$ are the principal curvatures at vertex v. To compute the principal curvatures we follow the method introduced by Ramakrishnan et al. [18, 19]. The self avoidance of surface is satisfied by setting the link length l as $a_0 \leq l \leq \sqrt{3}a_0$ where a_0 is the hard sphere radius of the vertices.

In DTMC the membrane surface is equilibrated through two independent Monte Carlo moves namely vertex moves and link flips. In a vertex move the position X_v of a vertex is updated to a new random position $X_v + \delta X_p$ within a cubic box of size ϵ_v. The size of the box is selected such that 50% of moves are accepted. This move simulates the thermal fluctuations and allows the membrane shape to relax to an equilibrium conformation. In a link flip move: a randomly selected link, between two triangles is disconnected, and new a link is established with the unconnected vertices of the same two triangles. This move makes the triangulation dynamic and preserves the fluid nature of the bilayer membranes by ensuring the in-plane displacement of vertices. MC moves are accepted through the Metropolis algorithm.

Ellipsoidal model for GNR: In our representation the GNR is modeled as an ellipsoid with three principal dimensions: $a = 70\,\text{nm}$, $b = c = 20\,\text{nm}$. The surface of the ellipsoid is discretized into 162 equally spaced points. The discrete points on the model GNR surface interact with membrane vertices via a truncated Lennard Jones potential given as

$$V_{LJ}(r) = 4\epsilon_{LJ}\left[\left(\frac{\sigma}{r}\right)^{12} - \left(\frac{\sigma}{r}\right)^{6}\right]\begin{cases}1 \text{ if } r \leq r_t,\\ S(r) \text{ if } r_t \leq r \leq r_c,\end{cases} \tag{S24}$$

where r is the distance of the dimer spheres from the membrane vertices and $S(r)$ is a function to smoothly truncate

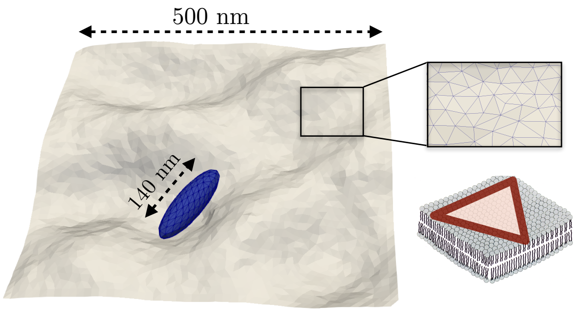

FIG. S10. A snapshot of triangulated surface membrane with a bound GNR.

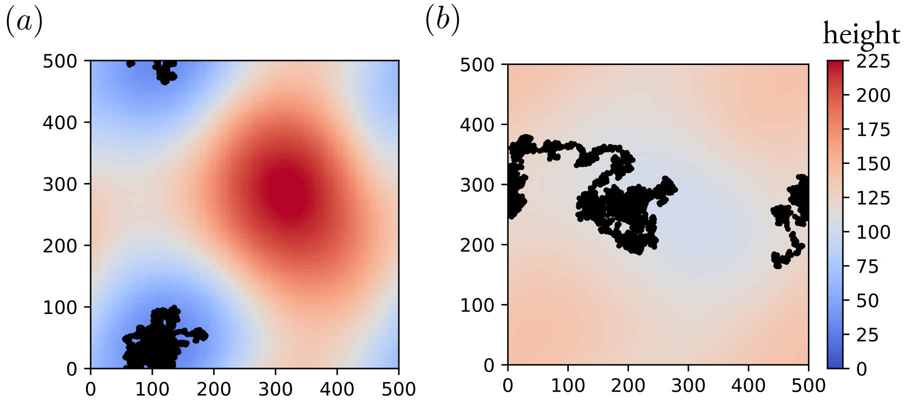

FIG. S11. Trajectory of model GNR on membrane surface at (a) high and (b) low $A_{\rm ex}$ for a time span of 2.5×10^6 MC steps.

the potential, given as

$$S(r) = 1 - \frac{(r - r_t)^2(3r_c - 2r - r_t)}{(r_c - r_t)^3}. \tag{S25}$$

We choose $r_t = 1.1688\sigma$ and $r_c = 1.3636\sigma$. We choose $\sigma = 15\,\text{nm}$ and $\epsilon_{LJ} = 2.5\ k_BT$. A snapshot of GNR-membrane model is given in Fig. S10.

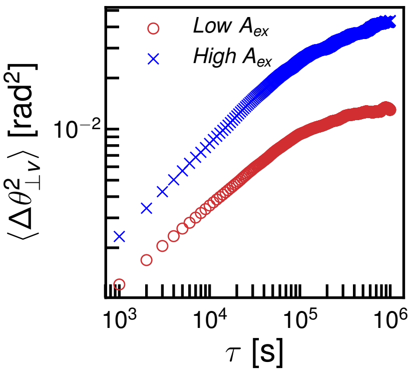

FIG. S12. Mean squared angular deviation of $\Delta\theta_{\perp v}$ where $\Delta\theta_{\perp v} = \cos^{-1}\left(\frac{1}{n}\sum_v \hat{n_t^v}.\hat{z}\right) - \cos^{-1}\left(\frac{1}{n}\sum_v \hat{n_0^v}.\hat{z}\right)$, n runs over all membrane vertices that are attached to GNR and $\hat{n}_t^v$ is the membrane vertex normal.

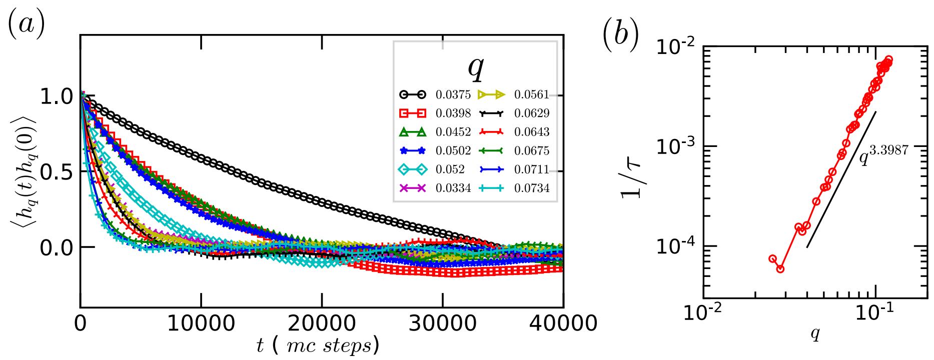

FIG. S13. (a) Membrane height correlation in frequency space. (b) Relaxation rate as a function of q obtained by fitting the correlation curve in (a) to exponential.

$A_{ex}(\%)$	$\sigma(k_BT/nm^2)$	$D(nm^2/\text{mcs steps})$
2.3	0.0216	0.0069
3.3	0.0191	0.0058
4.5	0.0173	0.0055
16.1	0.0172	0.0029
20.0	0.0166	0.0031
21.3	0.0153	0.0031

TABLE S2. Membrane tension and ellipsoidal nanoparticle diffusion coefficient as a function of A_{ex}.

S8. GNR MOTION ON A PLASMA MEMBRANE

A 2D trajectory of the GNR moving on the plasma membrane is shown in Fig. S14. The GNR performs 2D random walk on the membrane and stays in focus for more than 5 minutes. The azimuthal and polar angles of the nanorod and its orientation vector, $\hat{\boldsymbol{u}}$, and the reconstructed normal vector of the plasma membrane, $\hat{\boldsymbol{n}}$, are shown in Fig. S15.

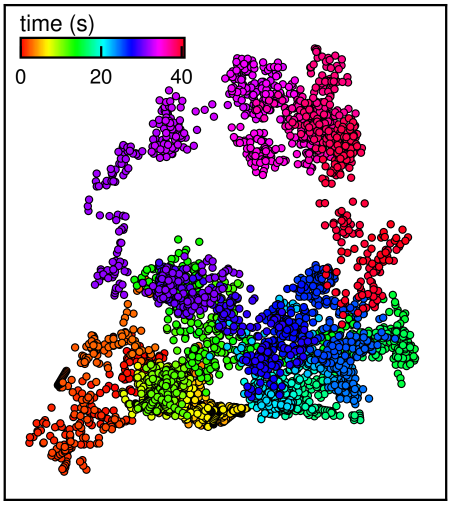

FIG. S14. Trajectory of the GNR diffusing randomly on the plasma membrane of a Huh7 cell.

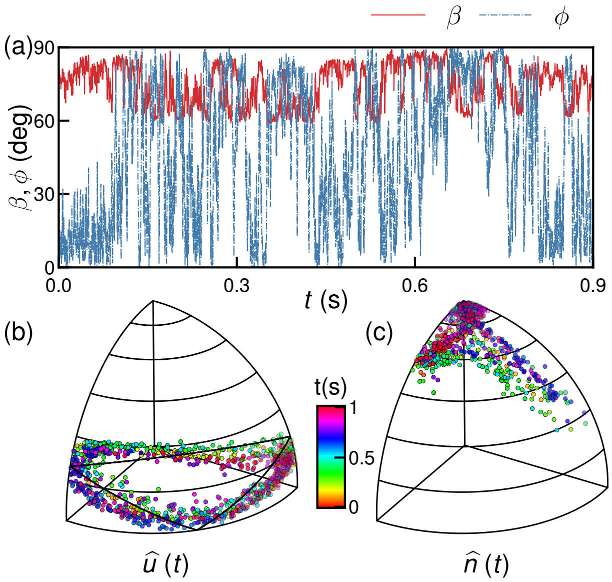

FIG. S15. Motion of a GNR on a plasma membrane of a Huh7 cell. a) Azimuthal, ϕ, and polar, β, angles. b) GNR orientation, $\hat{\boldsymbol{u}}$, indicates that it is limited to 2D plane which slowly fluctuates. c) The normal vector of the plasma membrane, $\hat{\boldsymbol{n}}$ estimated from $\hat{\boldsymbol{u}}$.

S9. VIDEOS

Video S1: Random rotational motion of a rod on a tilted plane.

Video S2: The simulated motion of a nanorod on simulated membranes with two different values of excess membrane area, corresponding to the tense and floppy membranes.

S10. CODE REPOSITORY

MATLAB analysis routines for GNR tracking and fitting procedure will be available on GitHub (https://github.com/mehdimolaei/NRtracking)

[1] W. Xie, L. Wang, Y. Zhang, L. Su, A. Shen, J. Tan, and J. Hu, Nuclear Targeted Nanoprobe for Single Living Cell Detection by Surface-Enhanced Raman Scattering, Bioconjugate Chemistry **20**, 768 (2009).

[2] M. I. Angelova and D. S. Dimitrov, Liposome electroformation, Faraday Discussions of the Chemical Society **81**, 303 (1986).

[3] A. Tian and T. Baumgart, Sorting of Lipids and Proteins in Membrane Curvature Gradients, Biophysical Journal **96**, 2676 (2009).

[4] M. Molaei, E. Atefi, and J. C. Crocker, Nanoscale Rheology and Anisotropic Diffusion Using Single Gold Nanorod Probes, Physical Review Letters **120**, 118002 (2018).

[5] A. J. Levine, T. B. Liverpool, and F. C. MacKintosh, Dynamics of rigid and flexible extended bodies in viscous films and membranes, Physical Review Letters **93**, 038102 (2004).

[6] P. G. Saffman and M. Delbrück, Brownian motion in biological membranes, Proceedings of the National Academy of Sciences **72**, 3111 (1975).

[7] T. T. Hormel, S. Q. Kurihara, M. K. Brennan, M. C. Wozniak, and R. Parthasarathy, Measuring lipid membrane viscosity using rotational and translational probe diffusion, Physical Review Letters **112**, 188101 (2014).

[8] M. Khan and T. G. Mason, Random walks of colloidal probes in viscoelastic materials, Physical Review E **89**, 042309 (2014), publisher: American Physical Society.

[9] W. Helfrich, Elastic Properties of Lipid Bilayers: Theory and Possible Experiments, Zeitschrift für Naturforschung C **28**, 693 (1973).

[10] U. Seifert and S. A. Langer, Viscous Modes of Fluid Bilayer Membranes, Europhysics Letters (EPL) **23**, 71 (1993).

[11] M. Mell, L. H. Moleiro, Y. Hertle, I. López-Montero, F. J. Cao, P. Fouquet, T. Hellweg, and F. Monroy, Fluctuation dynamics of bilayer vesicles with intermonolayer sliding: Experiment and theory, Chemistry and Physics of Lipids **185**, 61 (2015).

[12] R. Rodríguez-García, L. R. Arriaga, M. Mell, L. H. Moleiro, I. López-Montero, and F. Monroy, Bimodal Spectrum for the Curvature Fluctuations of Bilayer Vesicles: Pure Bending plus Hybrid Curvature-Dilation Modes, Physical Review Letters **102**, 128101 (2009).

[13] M. C. Watson, E. G. Brandt, P. M. Welch, and F. L. H. Brown, Determining Biomembrane Bending Rigidities from Simulations of Modest Size, Physical Review Letters **109**, 028102 (2012).

[14] U. Seifert, Configurations of fluid membranes and vesicles, Advances in Physics **46**, 13 (1997).

[15] N. Morone, T. Fujiwara, K. Murase, R. S. Kasai, H. Ike, S. Yuasa, J. Usukura, and A. Kusumi, Three-dimensional reconstruction of the membrane skeleton at the plasma membrane interface by electron tomography, Journal of Cell Biology **174**, 851 (2006).

[16] A. Kusumi, C. Nakada, K. Ritchie, K. Murase, K. Suzuki, H. Murakoshi, R. S. Kasai, J. Kondo, and T. Fujiwara, Paradigm Shift of the Plasma Membrane Concept from the Two-Dimensional Continuum Fluid to the Partitioned Fluid: High-Speed Single-Molecule Tracking of Membrane Molecules, Annual Review of Biophysics and Biomolecular Structure **34**, 351 (2005).

[17] J. S. Ho and A. Baumgärtner, Self-avoiding tethered membranes, Physical Review Letters **63**, 1324 (1989).

[18] N. Ramakrishnan, P. B. Sunil Kumar, and J. H. Ipsen, Monte Carlo simulations of fluid vesicles with in-plane orientational ordering, Physical Review E **81**, 041922 (2010).

[19] N. Ramakrishnan, K. K. Sreeja, A. Roychoudhury, D. M. Eckmann, P. S. Ayyaswamy, T. Baumgart, T. Pucadyil, S. Patil, V. M. Weaver, and R. Radhakrishnan, Excess area dependent scaling behavior of nano-sized membrane tethers, Physical Biology **15**, 026002 (2018).
